# A Double-Blind, Randomized, Placebo-Controlled, Phase II Clinical Study To Evaluate the Efficacy and Safety of Camostat Mesylate (DWJ1248) in Adult Patients with Mild to Moderate COVID-19

**DOI:** 10.1128/aac.00452-22

**Published:** 2022-12-14

**Authors:** Yeon-Sook Kim, Seng-Ho Jeon, Junghee Kim, Jong Hoon Koh, Seung Won Ra, Ji Won Kim, Yeonjae Kim, Choon Kwan Kim, Yun Chul Shin, Beo Deul Kang, Seung ji Kang, Chul Hee Park, Boyoung Lee, Ji Yeon Lee, Chung Hoon Lee, Jae-phil Choi, Jin Yong Kim, Shi Nae Yu, Kyong Ran Peck, Sung-Han Kim, Jung Yeon Heo, Hyun ah Kim, Hyun-jin Park, Jongwon Choi, Jumi Han, JooHyun Kim, Hyoung jun Kim, Se Hee Han, Aeri Yoon, MiHee Park, SuJung Park, YuKyung Kim, Minji Jung, Myoung-don Oh

**Affiliations:** a Department of Internal Medicine, Chungnam National University School of Medicine, Daejeon, South Korea; b Daewoong Pharmaceutical Co., Ltd., Seoul, South Korea; c Department of Neurosurgery, Seoul Medical Center, Seoul, South Korea; d Department of Internal Medicine, Seonam Hospital, Seoul, South Korea; e Department of Internal Medicine, Ulsan University Hospital, University of Ulsan College of Medicine, Ulsan, South Korea; f Department of Internal Medicine, SMG-SNU Boramae Medical Center, Seoul National University College of Medicine, Seoul, South Korea; g Division of Infectious Diseases, Department of Internal Medicine, National Medical Center, Seoul, South Korea; h Department of Internal Medicine, Veterans Health Service Medical Center, Seoul, South Korea; i Department of Anesthesiology and Pain Medicine, Gyeonggi Provincial Medical Center Pocheon Hospital, Pocheon, South Korea; j Department of Internal Medicine, Gyeonggi Provincial Medical Center Ansung Hospital, Ansung, South Korea; k Department of Internal Medicine, Chonmam National University Medical School, Gwangju, South Korea; l Department of Internal Medicine, Gyeonggi Provincial Medical Center Icheon Hospital, Icheon, South Korea; m Department of Internal Medicine, Gyeonggi Provincial Medical Center Uljeongbu Hospital, Uljeongbu, South Korea; n Department of Internal Medicine, Keimyung University Dongsan Hospital, Daegu, South Korea; o Department of Obstetrics and Gynecology, Gyeonggi Provincial Medical Center Suwon Hospital, Suwon, South Korea; p Department of Internal Medicine, Seoul Medical Center, Seoul, South Korea; q Department of Internal Medicine, Incheon Medical Center, Incheon, South Korea; r Department of Internal Medicine, Soonchunhyang University Cheonan Hospital, Cheonan, South Korea; s Division of Infectious Diseases, Samsung Medical Center, Sungkyunkwan University School of Medicine, Seoul, South Korea; t Department of Internal Medicine, Asan Medical Center, Seoul, South Korea; u Department of Infectious Diseases, Ajou University School of Medicine, Suwon, South Korea; v Department of Medicine, Division of Infectious Diseases, Keimyung University Dongsan Hospital, Keimyung University School of Medicine, Daegu, South Korea; w Clinical Development Center, Daewoong Pharmaceutical Co., Ltd., Seoul, South Korea; x Department of Internal Medicine, Seoul National University College of Medicine, Seoul, South Korea

**Keywords:** coronavirus disease 2019, SARS-CoV-2, antiviral agent, camostat mesylate, clinical trial

## Abstract

Although several antiviral agents have become available for coronavirus disease 2019 (COVID-19) treatment, oral drugs are still limited. Camostat mesylate, an orally bioavailable serine protease inhibitor, has been used to treat chronic pancreatitis in South Korea, and it has an *in vitro* inhibitory potential against severe acute respiratory syndrome coronavirus 2 (SARS-CoV-2). This study was a double-blind, randomized, placebo-controlled, multicenter, phase 2 clinical trial in mild to moderate COVID-19 patients. We randomly assigned patients to receive either camostat mesylate (DWJ1248) or placebo orally for 14 days. The primary endpoint was time to clinical improvement of subject symptoms within 14 days, measured using a subjective 4-point Likert scale. Three hundred forty-two patients were randomized. The primary endpoint was nonsignificant, where the median times to clinical improvement were 7 and 8 days in the camostat mesylate group and the placebo group, respectively (hazard ratio [HR] = 1.09; 95% confidence interval [CI], 0.84 to 1.43; *P* = 0.50). A *post hoc* analysis showed that the difference was greatest at day 7, without reaching significance. In the high-risk group, the proportions of patients with clinical improvement up to 7 days were 45.8% (50/109) in the camostat group and 38.4% (40/104) in the placebo group (odds ratio [OR] = 1.33; 95% CI, 0.77 to 2.31; *P* = 0.31); the ordinal scale score at day 7 improved in 20.0% (18/90) of the camostat group and 13.3% (12/90) of the placebo group (OR = 1.68; 95% CI, 0.75 to 3.78; *P* = 0.21). Adverse events were similar in the two groups. Camostat mesylate was safe in the treatment of COVID-19. Although this study did not show clinical benefit in patients with mild to moderate COVID-19, further clinical studies for high-risk patients are needed. (This trial was registered with ClinicalTrials.gov under registration no. NCT04521296).

## INTRODUCTION

Since its emergence in December 2019, coronavirus disease 2019 (COVID-19) has spread worldwide. As of 9 February 2022, the cumulative number of COVID-19 cases was over 399 million, including more than 5.8 million deaths (1). Although several therapeutic agents for the treatment of COVID-19 have become available (2–7), oral antiviral agents like molnupiravir and nirmatrelvir/ritonavir against severe acute respiratory syndrome coronavirus 2 (SARS-CoV-2) are urgently needed.

Camostat mesylate (DWJ1248, Foistar) is an orally bioavailable synthetic serine protease inhibitor and a strong inhibitor of trypsin, plasmin, plasma kallikrein, and thrombin (8). It was first developed and licensed in Japan in 1985 and has been used for the treatment of acute exacerbation of chronic pancreatitis and reflux esophagitis in Japan and South Korea (9, 10). As a potent inhibitor of transmembrane protease serine type 2 (TMPRSS2), camostat mesylate can inhibit cell membrane fusion of respiratory viruses, such as influenza virus and coronaviruses (11). *In vitro* and animal model studies show that it can inhibit the replication of influenza viruses (11, 12). It also inhibits the replication of coronaviruses, including severe acute respiratory syndrome coronavirus (SARS-CoV), Middle East respiratory syndrome coronavirus (MERS-CoV), human coronavirus 229E (HCoV-229E), and SARS-CoV-2 (11, 13–15).

According to the literature, an *in vitro* study was first conducted as a prior study, suggesting that camostat mesylate would be effective (16). Consequently, an *in vitro* study using the lung-derived human cell line Calu-3 and vesicular stomatitis virus reporter particles pseudotyped with SARS-CoV-2 spike showed that a 50% effective concentration of camostat mesylate was 87 nM (14). This target is theoretically achievable at this level, after a loading dose of 200 mg, and unlikely to be maintained after one half-life.

Camostat mesylate is a promising candidate for the treatment of COVID-19, as it is orally bioavailable, affordable, and widely available. To explore the possibility of clinical benefit of camostat mesylate in the treatment of COVID-19, we conducted a randomized, placebo-controlled, phase 2 clinical trial in adult patients with mild to moderate COVID-19 (ClinicalTrials.gov registration no. NCT04521296).

## RESULTS

**FIG 1:**
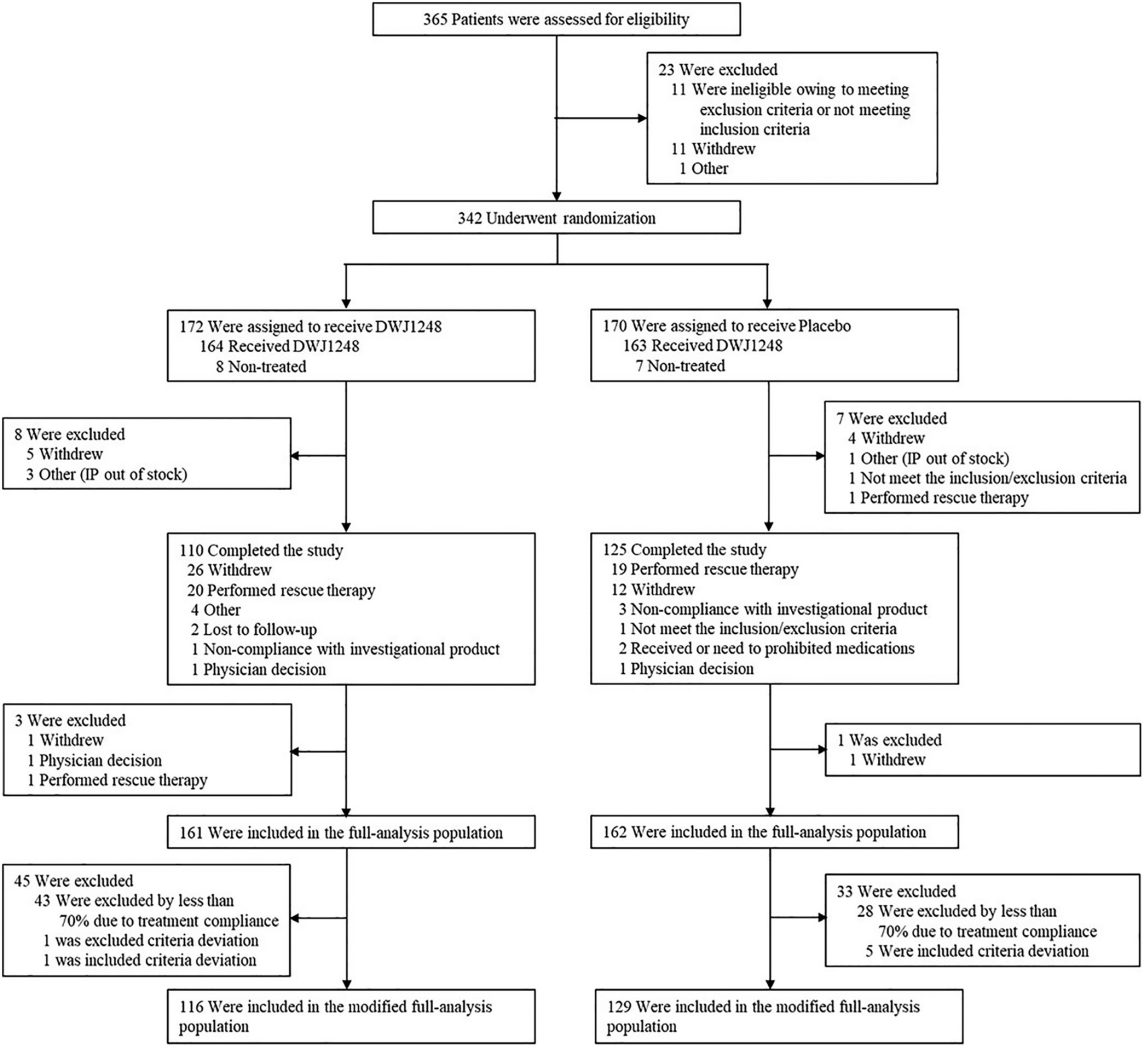
Enrollment and randomization.

A total of 365 patients from 21 institutions in South Korea were assessed between February and May 2021, and 342 patients were enrolled in the study (Fig. 1). After randomization, 172 were assigned to receive camostat mesylate (camostat group) and 170 were assigned to receive placebo (placebo group). Of the 172 patients in the camostat group, 62 dropped out of the study (31 due to withdrawal by subject, 1 due to noncompliance with the investigational product [IP], 2 due to loss to follow up, 1 due to withdrawal by the investigator, and 7 due to other causes), and of the 170 patients in the placebo group, 45 dropped out of the study (2 due to ineligibility for the study, 16 due to withdrawal by subject, 3 due to noncompliance with the investigational product, 2 due to prohibited medication/therapies, 1 due to withdrawal by the investigator, and 1 due to other causes). Hence, out of 342 enrolled patients, 235 patients completed this study. The main reason for early termination was withdrawal of consent by 47 patients because of their reluctance to be monitored continuously after discharge.

Of the 342 randomized patients, a total of 323 patients (161 in the camostat group and 162 in the placebo group), excluding 15 who did not receive investigational products and 4 who missed the “Efficacy Variables Check,” were included in the full analysis set (FAS). A total number of 245 patients (116 in the camostat group and 129 in the placebo group), excluding 78 patients from the FAS (6 due to “Inclusion Criteria Deviation,” 1 due to “Exclusion Criteria Deviation,” and 71 due to “IP Handling and Administration”), was included in the modified full analysis set (mFAS). The median age was 53.0 years in the camostat group and 53.5 years in the placebo group, and 53.22% (182/342) were male. In all, 54.39% (186/342) had one or more risk factors, such as cardiovascular disease, chronic respiratory disease, high blood pressure, diabetes, obesity, and smoking, of which 55.81% (96/172) were in the camostat group and 52.94% (90/170) in the placebo group. At baseline, 72.22% (247/342) had a mild and 27.78% (95/342) had a moderate severity of COVID-19 (Table 1). Two hundred twenty-eight patients were in the high-risk group, and the median age was 60.5 years for both camostat and placebo groups. Of the patients with high-risk factors, 12.90% (24/186) had cardiovascular disease, 1.61% (3/186) had chronic respiratory disease, 55.91% (104/186) had high blood pressure, 25.27% (47/228) had diabetes, 14.52% (27/186) had obesity, and 26.88% (50/228) were smokers; there was no significant difference between the camostat and placebo groups (Table S1 in the supplemental material).

**TABLE 1:** Demographics and baseline characteristics of the randomized set

Parameter^*a*^	Value [no. (%) or as indicated] for group^*b*^		
	DWJ1248 (*n* = 172)	Placebo (*n* = 170)	Total (*n* = 342)
Age (yrs)			*P* = 0.4715 (w)^*c*^
Mean (SD)	52.15 (14.55)	50.68 (15.14)	51.42 (14.84)
Median (min, max)	53.00 (22.00, 81.00)	53.50 (19.00, 79.00)	53.00 (19.00, 81.00)
Age group			*P* = 0.8888 (c)^*d*^
≥60 yrs	65 (37.79)	63 (37.06)	128 (37.43)
Sex			*P* = 0.4523 (c)^*d*^
Male	95 (55.23)	87 (51.18)	182 (53.22)
Female	77 (44.77)	83 (48.82)	160 (46.78)
Race			
American Indian or Alaska Native	0	0	0
Asian	172 (100.00)	170 (100.00)	342 (100.00)
Black or African American	0	0	0
Native Hawaiian or Other Pacific Islander	0	0	0
White	0	0	0
Other	0	0	0
Ethnicity			
Hispanic or Latino	0	0	0
Not Hispanic or Latino	172 (100.00)	170 (100.00)	342 (100.00)
Unknown	0	0	0
BMI (kg/m^2^)			*P* = 0.8982 (w)^*c*^
Mean (SD)	24.95 (3.66)	24.87 (3.76)	24.91 (3.71)
Median (min, max)	24.50 (18.00, 41.20)	24.60 (16.30, 39.90)	24.50 (16.30, 41.20)
Smoker			*P* = 0.8833 (f)^*d*^
Nonsmoker	118 (68.60)	119 (70.00)	237 (69.30)
Smoker	25 (14.53)	25 (14.71)	50 (14.62)
Ex-smoker	29 (16.86)	25 (14.71)	54 (15.79)
Unknown	0	1 (0.59)	1 (0.29)
Risk factor			*P* = 0.5938 (c)^*d*^
Total	96 (55.81)	90 (52.94)	186 (54.39)
Cardiovascular disease	13 (13.54)^*e*^	11 (12.22)	24 (12.90)
Chronic respiratory disease	1 (1.04)	2 (2.22)	3 (1.61)
High blood pressure	50 (52.08)	54 (60.00)	104 (55.91)
Diabetes	28 (29.17)	19 (21.11)	47 (25.27)
Obesity	14 (14.58)	13 (14.44)	27 (14.52)
Smoking	25 (26.04)	25 (27.78)	50 (26.88)
COVID-19 severity at baseline			*P* = 0.1017 (c)^*d*^
Mild	131 (76.16)	116 (68.24)	247 (72.22)
Moderate	41 (23.84)	54 (31.76)	95 (27.78)
Other	0	0	0
COVID-19 antibody			*P* = 0.4943 (f)^*d*^
Total	32	36	68
Yes	0	2 (5.56)^*f*^	2 (2.94)
Age group/risk factor			*P* = 0.7597 (c)^*d*^
≥60 yrs or risk factor: yes	116 (67.44)	112 (65.88)	228 (66.67)
Subjective symptoms at baseline			*P* = 0.4469 (c)^*d*^
Total	169	167	336
At least one moderate or severe	60 (35.50)	66 (39.52)	126 (37.50)
Other	109 (64.50)	101 (60.48)	210 (62.50)
RT-PCR at baseline			*P* = 0.4183 (f)^*d*^
Negative	6 (3.49)	3 (1.76)	9 (2.63)
Positive	165 (95.93)	167 (98.24)	332 (97.08)
Inconclusive	1 (0.58)	0	1 (0.29)
Ordinal scale at baseline			*P* = 0.4913 (f)^*d*^
0	4 (2.33)	2 (1.18)	6 (1.75)
1	0	0	0
2	74 (43.02)	66 (38.82)	140 (40.94)
3	94 (54.65)	102 (60.00)	196 (57.31)
NEWS score at baseline			*P* = 0.9195 (f)^*d*^
0	71 (41.28)	62 (36.47)	133 (38.89)
1	59 (34.30)	64 (37.65)	123 (35.96)
2	27 (15.70)	25 (14.71)	52 (15.20)
3	10 (5.81)	12 (7.06)	22 (6.43)
4	4 (2.33)	6 (3.53)	10 (2.92)
5	1 (0.58)	1 (0.59)	2 (0.58)
NEWS severity at baseline			*P* = 0.3699 (f)^*d*^
Mild	171 (99.42)	167 (98.24)	338 (98.83)
Moderate	1 (0.58)	3 (1.76)	4 (1.17)
Severe	0	0	0

a Min, minimum; max, maximum; BMI, body mass index; NEWS, national early warning score.

d See the description of the randomized set in Results. For the data below, unless otherwise indicated, the denominator of the percentage is the number of subjects in each group.

c Testing for difference between DWJ1248 and placebo using two-sample *t* test (t) or Wilcoxon rank sum test (w).

d Testing for difference between DWJ1248 and placebo using chi-square test (c) or Fisher’s exact test (f).

e The denominator of the percentage for individual risk factors is the number of subjects with the risk factor in each group.

f The denominator of the percentage for COVID-19 antibody is the number of subjects who had a positive result for COVID-19 antibody in each group.

**FIG 2:**
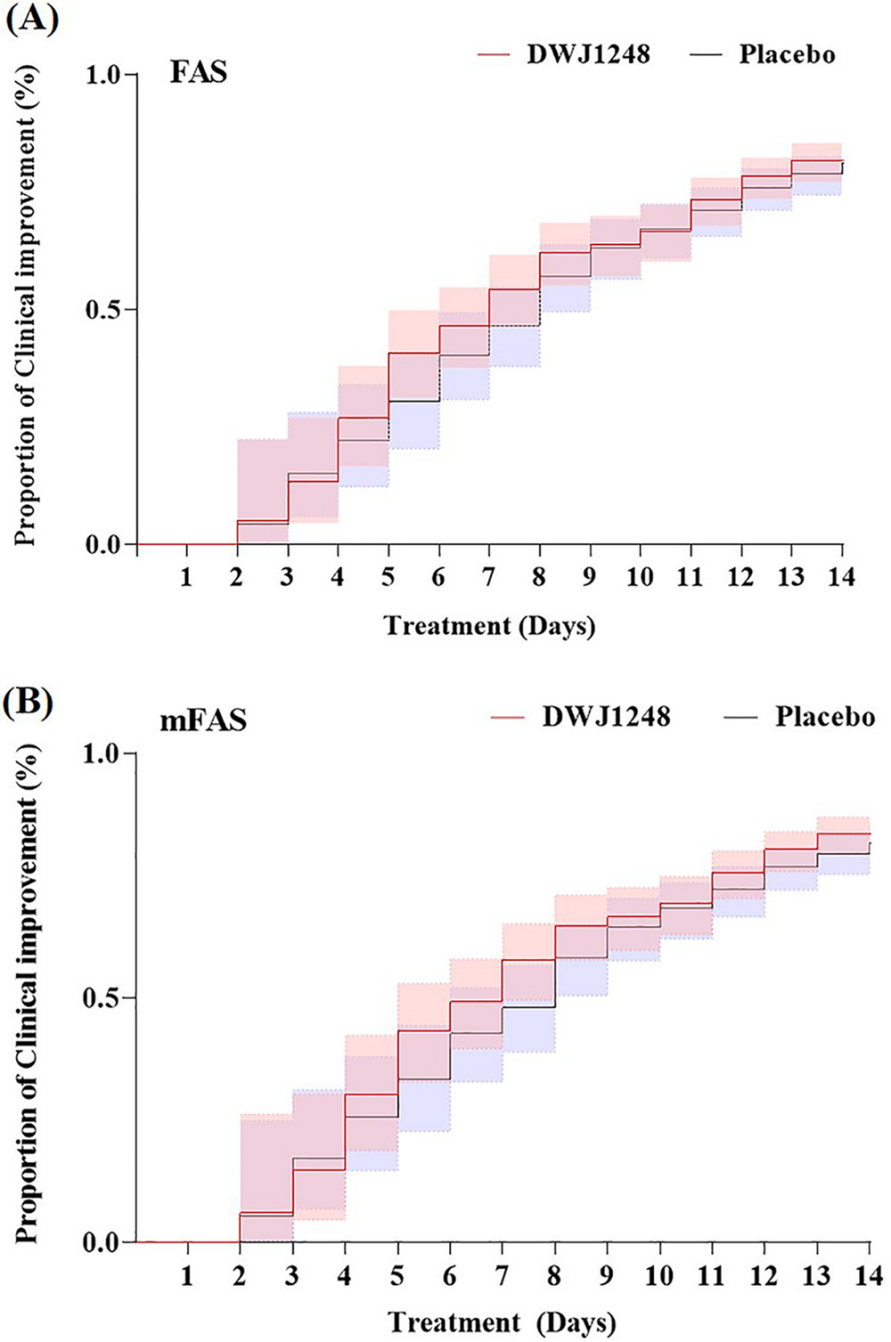
Kaplan-Meier plot for time to clinical improvement of subjective symptoms.

The primary endpoint, time to clinical improvement of subject symptoms within 14 days, was not significantly different between the two groups, being 7 days in the camostat group and 8 days in the placebo group (95% confidence interval [CI], 0.84 to 1.43; *P* = 0.50) (Table 2). A Kaplan-Meier plot for times to clinical improvement in the FAS and mFAS showed a trend of faster clinical improvement in the camostat group, especially from day 4 to day 10 (Fig. 2).

**TABLE 2:** Clinical improvement in subjective symptoms in the FAS^*a*^

Parameter^*b*^	Value for group^*c*^	
	DWJ1248 (*n* = 161)	Placebo (*n* = 162)
Clinical improvement status^*d*^		
No. (%) of subjects with clinical improvement (event)	107 (66.46)	115 (70.99)
95% CI	59.17–73.75	64.00–77.98
Time (days) to clinical improvement in subjective symptoms by day 14^*d*^		
Mean (SD)	5.94 (2.93)	6.73 (3.32)
Median	5.00	6.00
Min, max	2.00, 13.00	2.00, 15.00
Median (95% CI)^*g*^	7.00 (6.00–8.00)	8.00 (7.00–9.00)
*P* value^*e*^	0.4951	
Hazard ratio	1.09	
95% CI	0.84–1.43	
*P* value^*f*^	0.5035	

a FAS, full analysis set. See the description of the FAS in Results.

b Min, minimum; max, maximum; CI, confidence interval.

c The denominator of the percentage is the number of subjects who have symptoms at baseline for each group.

d Duration (days) = (date of event/censored, whichever occurs first − date of first IP administration +1); status = event (clinical improvement) if there is clinical improvement at least once by day 14, and status = censored (no clinical improvement) if there is no improvement by day 14 but clinical improvement after day 14 or no improvement by the end of the study or there is rescue therapy usage before clinical improvement by day 14.

e Testing for difference between DWJ1248 and placebo using the log rank test.

f Testing for difference between DWJ1248 and placebo using the Cox proportional hazards model with treatment group as a factor and age and risk factor as covariates. If the result or CI was estimated to be infinity, hazard ratio was estimated using Firth’s penalized maximum likelihood and its CI was presented as the profile penalized likelihood confidence interval.

g The median time to clinical improvement and its 95% confidence interval by treatment group using the Kaplan-Meier curve.

In the FAS of the high-risk group, the proportions with improvement of subjective symptoms by day 7 were 45.87% (50/109) and 38.46% (40/104) in the camostat group and the placebo group, respectively (95% CI, 0.77 to 2.31; *P* = 0.31), while in the mFAS of the high-risk group, the proportions were 57.14% (44/77) and 41.03% (32/78) in the camostat group and the placebo group, respectively (95% CI, 0.97 to 3.59; *P* = 0.06) (Table 3).

**TABLE 3:** Clinical improvement of subjective symptoms in high-risk group in the FAS and the mFAS

Parameter	Value for^*a*^:			
	FAS		mFAS	
	DWJ1248 (*n* = 109)	Placebo (*n* = 104)	DWJ1248 (*n* = 77)	Placebo (*n* = 78)
Clinical improvement by day 7				
No. (%) of subjects^*b*^	50 (45.87)	40 (38.46)	44 (57.14)	32 (41.03)
95% CI	36.52–55.23	29.11–47.81	46.09–68.20	30.11–51.94
*P* value^*c*^	0.2738		0.0448	
Odds ratio^*d*^	1.33		1.86	
95% CI	0.77–2.31		0.97–3.59	
*P* value^*e*^	0.3065		0.0636	
Clinical improvement by day 14				
No. (%) of subjects^*b*^	67 (61.47)	70 (67.31)	61 (79.22)	61 (78.21)
95% CI	52.33–70.60	58.29–76.32	70.16–88.28	69.04–87.37
*P* value^*c*^	0.3738		0.8773	
Odds ratio^*d*^	0.78		1.05	
95% CI	0.44–1.37		0.48–2.31	
*P* value^*e*^	0.3840		0.8943	

a FAS, full analysis set; mFAS, modified full analysis set. See the descriptions of the FAS and the mFAS in Results.

b The denominator of the percentage is the number of subjects in each group.

c Testing for difference between treatment groups using the chi-square test.

d Odds ratio was estimated using Firth’s penalized maximum likelihood, and its CI was presented as the profile penalized likelihood confidence interval.

e Testing for difference between treatment groups using the logistic regression model with treatment group as a factor and age and risk factor as a covariate.

In the high-risk group, the ordinal scale score improved by day 7 in 20.00% (18/90) of the camostat group in the FAS, compared to 13.33% (12/90) of the placebo group (95% CI, 0.75 to 3.78; *P* = 0.21), while in the mFAS, the proportions were 22.08% (17/77) of the camostat group and 11.54% (9/78) of the placebo group (95% CI, 0.97 to 5.89, *P* = 0.06) (Table 4).

**TABLE 4:** Improvement in ordinal scale in high-risk group in the FAS and the mFAS

Parameter	Value for^*a*^:			
	FAS		mFAS	
	DWJ1248 (*n* = 109)	Placebo (*n* = 104)	DWJ1248 (*n* = 77)	Placebo (*n* = 78)
No. of subjects with ordinal scale of ≥2 at baseline	107	103	77	78
Proportion of subjects with improved ordinal scale at day 7	90	90	77	78
No. (%) of subjects^*b*^	18 (20.00)	12 (13.33)	17 (22.08)	9 (11.54)
95% CI	11.74–28.26	6.31–20.36	12.81–31.34	4.45–18.63
*P* value^*c*^	0.2301 (c)		0.0791 (c)	
Odds ratio^*d*^	1.68		2.39	
95% CI	0.75–3.78		0.97–5.89	
*P* value^*e*^	0.2088		0.0592	
Proportion of subjects with improved ordinal scale at day 14	73	81	71	75
No. (%) of subjects^*b*^	42 (57.53)	42 (51.85)	40 (56.34)	37 (49.33)
95% CI	46.20–68.87	40.97–62.73	44.80–67.87	38.02–60.65
*P* value^*c*^	0.4795 (c)		0.3968 (c)	
Odds ratio^*d*^	1.33		1.43	
95% CI	0.69–2.57		0.73–2.82	
*P* value^*e*^	0.3948		0.3000	

a FAS, full analysis set; mFAS, modified full analysis set. See the descriptions of the FAS and the mFAS in Results.

b The denominator of the percentage is the number of subjects in each group.

c Testing for difference between treatment groups using the chi-square test.

d Odds ratio was estimated using Firth’s penalized maximum likelihood, and its confidence interval (CI) is presented as the profile penalized likelihood confidence interval.

e Testing for difference between treatment groups using the logistic regression model with treatment group as a factor and age and risk factor as a covariate.

**FIG 3:**
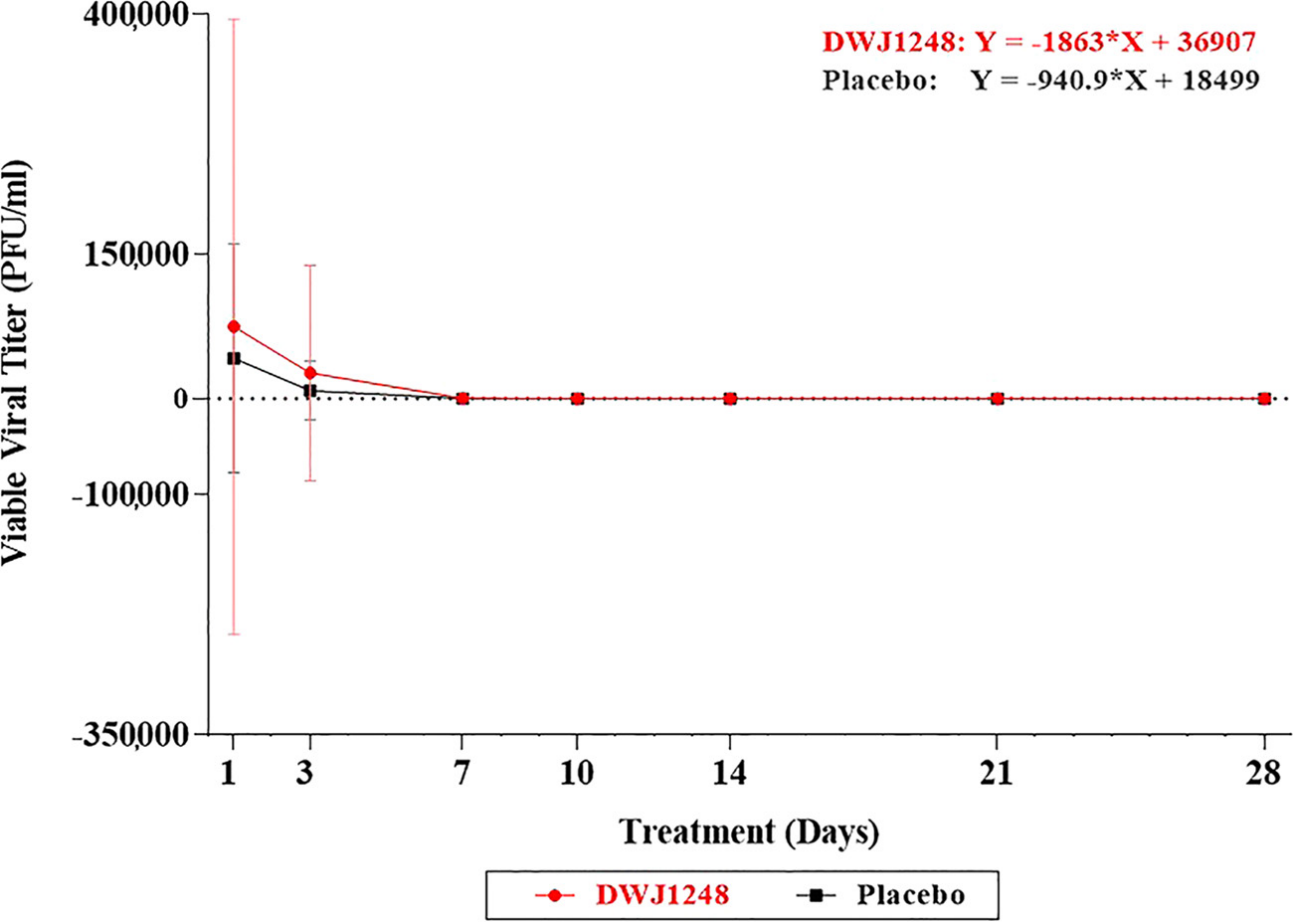
Virus culture using plaque assay in SARS-CoV-2-infected patient samples. Error bars show standard deviations.

Virus culture was previously planned as an exploratory endpoint in this study’s protocol. The virus culture test was performed on swab samples obtained from the nasopharynx. For the quantification of SARS-CoV-2, nasopharyngeal swab samples were taken at predetermined intervals. All the clinical samples were transferred to a single central laboratory and were analyzed there. SARS-CoV-2 culture was performed and viral titers were assessed as described previously (17). The amount of live virus decreased more rapidly in the camostat group than in the placebo group. The result of linear regression of the amount of virus (PFU/mL) demonstrated that the camostat group had a slope of 1,446 and the placebo group had a slope of 659.6 (Fig. 3). After day 7, the virus did not grow in most samples taken from the patients of both groups. However, no statistically significant results could be obtained.

Concomitant medications (Piroxicam patch and Diphenhydramine Hydrochloride patch) used included analgesics, antibiotics, antihistamine, nonsteroidal anti-inflammatory drugs (NSAIDs), cough medicine, sore throat medicine, muscle relaxant, patch, migraine reliever, and analgesic or cough medicine. The proportions of patients receiving the concomitant medications were not significantly different in the two groups, supporting the conclusion that taking camostat mesylate contributed to the improvement in patients’ clinical symptoms, rather than taking other standard-of-care medications (Table S2). Although not statistically different, 60.47% (104/172) of the camostat group and 68.82% (117/170) of the placebo group received cough medicine (95% CI, 59.55 to 69.69; *P* = 0.11).

The rates of incidence of treatment emergent adverse events (TEAEs) and adverse drug reactions (ADRs) were not statistically different between the camostat and placebo groups (Table 5): 57.93% (95/164) of the camostat group and 61.96% (101/163) of the placebo group had TEAEs (95% CI, 54.63 to 65.25; *P* = 0.46) and 5.45% (9/164) of the camostat group and 4.29% (7/163) of the placebo group experienced ADRs (95% CI, 2.55 to 7.23; *P* = 0.62). No serious adverse drug reactions were reported during the study period.

**TABLE 5:** Incidence of TEAEs and ADRs (safety set)

Parameter	No. (%) of subjects [no. of events] or 95% CI^*a*^		
	DWJ1248 (*n* = 164)	Placebo (*n* = 163)	Total (*n* = 327)
TEAEs	95 (57.93) [229]	101 (61.96) [198]	196 (59.94) [427]
95% CI	50.37–65.48	54.51–69.42	54.63–65.25
*P* value^*b*^			0.4564

ADRs	9 (5.49) [11]	7 (4.29) [8]	16 (4.89) [19]
95% CI	2.00–8.97	1.18–7.41	2.55–7.23
*P* value^*b*^			0.6170
			
No. of subjects with serious TEAEs	0	0	0
No. of subjects with serious ADRs	0	0	0

a The denominator of the percentage is the number of subjects in each group.

b Testing for difference between treatment groups using the chi-square test.

## DISCUSSION

In this phase 2 clinical trial, we assessed the clinical benefit and safety of camostat mesylate in the treatment of mild to moderate COVID-19. No primary or secondary endpoint was statistically significant, according to the *post hoc* analysis performed. Although the difference was not statistically significant, time to clinical improvement was 7 days in the camostat group, 1 day shorter than in the placebo group. In the high-risk group, the clinical severity score and ordinal scale score improved faster in the camostat group than in the placebo group. The amount of live virus decreased more rapidly in the camostat group.

A recent randomized, placebo-controlled clinical trial showed that camostat mesylate was not effective in clinical improvement, disease progression, or reduction of mortality. The median times to clinical improvement were 5 days in the camostat group and 5 days in the placebo group. The hazard ratio (HR) for 30-day mortality in the camostat group versus the placebo group was 0.82 (CI, 0.24 to 2.79). The median change in viral loads from baseline to day 5 in the camostat group was −0.22 log_10_ copies/mL (*P* < 0.05), and it was −0.82 log_10_ in the placebo group (*P* < 0.05) (18).

The RES Q-HR [Reconvalescent Plasma/Camostat Mesylate Early in SARS-CoV-2 Q-PCR (COVID-19) Positive High-risk Individuals] trial also aims to assess the effectiveness of camostat in prevention of disease progression in high-risk patients. It has recruited 22 patients, and its results are pending (19).

The COMOVID trial, another clinical trial for camostat mesylate in ambulatory adult patients, was terminated due to SARS-CoV2 pandemic evolution, with a decrease in inclusions and widespread distribution of vaccines (https://clinicaltrials.gov/ct2/show/record/NCT04608266?term=camostat&draw=2&rank=7).

Anticough medications were given to 134 patients: 60 patients in the camostat group and 74 patients in the placebo group (*P* = 0.03) (Table S2). Ambroxol and bromhexine were prescribed to 7 and 10 patients, respectively. Since these bronchial fluidizers were considered to have inhibitory properties toward TMPRSS2 (20), we attempted to make a further analysis. However, due to an insufficient number of patients who used cough medicine in this study, this specific bias could not be confirmed.

In this study, the efficacy of camostat mesylate was evaluated after administrating for 14 days and the safety profiles were monitored up to 28 days according to the requirement of the South Korea Ministry of Food and Drug Safety. The safety profile of patients who received camostat mesylate was similar to that of placebo-treated patients. In addition, the safety of camostat mesylate has been well established. Foistar has been on the market in South Korea and Japan for more than 2 decades as a treatment for chronic pancreatitis. In South Korea, over 22,400,000 tablets have been used from 2012 to 2020, but only 40 adverse events, 11 adverse drug reactions, and 5 serious adverse reactions have been reported (Korea Institute of Drug Safety & Risk Management, unpublished data). These data underscore the potential value of developing camostat mesylate as a safe, orally bioavailable, affordable, and widely available therapeutic agent for COVID-19.

Limitations of our study include insufficient power to detect anticipated differences in the primary endpoint; as this was a phase 2 clinical study, the number of participants was small. Our study results did not elucidate clinical improvement with camostat mesylate in mild and moderate COVID-19 patients. However, subgroup analysis of the high-risk group suggested clinical benefit. To further assess clinical efficacy of camostat mesylate, phase 3 clinical studies are needed.

## MATERIALS AND METHODS

### Study design.

This study was a double-blind, randomized, placebo-controlled, multicenter, phase 2 clinical trial in patients with mild to moderate COVID-19. Enrollment of participants began in February 2021 and ended in May 2021. There were 21 study sites, all in South Korea. This clinical trial was planned and recruited COVID-19 patients in South Korea in accordance with the quarantine guidelines, “Coronavirus Infectious Disease-19 Response Guidelines (10-2 edition)” (https://www.gne.go.kr/upload_data/board_data/BBS_0000902/163849322429842.pdf). In following South Korea’s quarantine guidelines at the time of the clinical trial, “hospitalization” was for patient isolation, not actual hospitalization due to the patient’s severity. In addition, during the period of this clinical trial in Korea, because all confirmed COVID-19 patients were hospitalized (isolated) at the hospitals or live-in treatment centers to receive treatment from medical staff, it was not possible to establish hospitalization rates as the efficacy endpoint as in other clinical trials. Participants were randomized 1:1 to the camostat mesylate or placebo arm. Camostat mesylate was administered orally at 200 mg three times a day for 14 days. A matching placebo was administered using the same dosing schedule as the active drug. All participants received supportive care according to the standard of care for the study site hospitals.

The study protocol was approved by the institutional review board at each study site. Written informed consent was obtained from all patients for participation in this study. This trial was registered with ClinicalTrials.gov (registration no. NCT04521296), and the study protocol can be seen in the supplemental material.

### Patients.

Participants who gave informed consent were assessed for eligibility. The inclusion criteria were age 19 years or older, real-time reverse transcription-PCR (RT-PCR)-confirmed SARS-CoV-2 infection, mild to moderate COVID-19, and one or more COVID-19 symptoms. Patients with mild COVID-19 had symptoms of COVID-19 but did not have signs of pneumonia on chest radiography; moderate COVID-19 was defined as subjects with pneumonia but without evidence of severe pneumonia; and severe pneumonia was defined as presenting oxygen saturation (SpO_2_) of less than 94% on room air or respiration rate of >30 breaths per minute at time of screening. The exclusion criteria were an inability to take oral medication; an alanine aminotransferase (ALT) or aspartate aminotransferase (AST) value of ≥5 times the upper limit of the normal range; impaired renal function (CrCl of <30 mL/min); a need for immunosuppressive agents; an HIV, hepatitis B virus (HBV), or hepatitis C virus (HCV) infection requiring antiviral treatment; and severe and critically severe patients. Severe patients were defined as subjects who had signs of pneumonia and needed supplemental oxygen therapy, such as nasal prong, facial mask, or high-flow oxygen therapy; critically severe patients were defined as subjects who needed noninvasive or invasive mechanical ventilation or extracorporeal membrane oxygen therapy (ECMO) or with acute respiratory distress syndrome (ARDS), shock, or multiple organ failure. In this clinical trial, patient recruitment was conducted from 1 February 2021 to 15 June 2021. During this period, the vaccine distribution was at the initial stage in Korea. Therefore, subjects who were vaccinated were not included in this study, as COVID-19 vaccine was a contraindicated drug in the clinical trial protocol.

### Outcome measures.

The primary endpoint was time (days) to clinical improvement of subjective symptoms within 14 days. A four-point scale (0, none; 1, mild; 2, moderate; and 3, severe) was utilized for subjective symptom assessment (21). Clinical improvement was defined as symptom severity of “none” or “mild” for at least 24 h in COVID-19 patients who had baseline symptoms (within 24 h prior to the first administration) of febrile sense, cough, shortness of breath, chills, muscle aches, headache, and sore throat (Fig. S1). Severity of clinical symptoms followed *Common Terminology Criteria for Adverse Events* (CTCAE) version 5.0 (22).

The secondary endpoints were time (days) to clinical improvement of subjective symptoms within 28 days, change from baseline in subjective symptom score, change from baseline in the ordinal scale, and proportion of clinical improvement in the ordinal scale. An 8-point ordinal scale was used in this study, where score 0 was for an uninfected patient state, 1 or 2 for ambulatory, 3 or 4 for hospitalized with mild disease, 5 to 7 for hospitalized with severe disease, and 8 for dead. An improved ordinal scale score was established when the ordinal scale score improved by 2 or more after the baseline among patients whose baseline ordinal scale score was 2 or higher.

Subgroup analysis of the high-risk group was conducted, where the high-risk group included patients who were older than 60 years or had one or more risk factors such as cardiovascular disease, chronic respiratory disease, high blood pressure, diabetes, obesity, and smoking.

The result of linear regression of the amount of virus (PFU/mL) was obtained using the lm equation of the R program (version 4.0.5).

### Randomization.

A stratified block randomization with age (<60 years versus ≥60 years) and the presence of risk factors (any versus none of cardiovascular disease, chronic respiratory disease, hypertension, diabetes, obesity, or smoking) as the stratification factors was used. Subject randomization codes were generated by an independent statistician using Statistical Analysis Software (SAS) version 9.4 (SAS Institute, Inc., Cary, NC, USA), and subjects were assigned to the treatment groups by the investigators via an interactive web-response system.

### Statistical analysis.

A total of 189 events across both groups was required, which would provide ≥89% power and a two-sided significance level of 5% if the hazard ratio (HR) comparing the camostat mesylate group to the placebo group was ≥1.6. Assuming a 70% probability of events within 28 days across both groups and a dropout rate of 20%, approximately 338 patients (169 patients per group) were determined for the sample size.

Efficacy was evaluated by full analysis set (FAS; based on the intention-to-treat principle) as the primary result and modified full analysis set (mFAS) as the secondary result. The FAS population was subjects who received at least 1 dose of the investigational product after randomization and had at least one assessment result for COVID-19 symptoms. The mFAS population was subjects included in the FAS whose investigational product (IP) compliance was 70% or higher without deviations from the inclusion/exclusion criteria.

The time to clinical improvement of subjective symptoms (days) was analyzed using the Kaplan-Meier curve and Cox proportional hazards regression model with a treatment group as the factor and stratification factors (age and presence of risk factors) as covariates. The proportion with improvement of subjective symptoms up to 7 days was analyzed using a logistic regression model with the treatment group as the factor and stratification factors (age and presence of risk factors) as covariates. For a sensitivity analysis, the log rank test or chi-square test was additionally performed.

Clinical data were collected and validated using the Rave Electronic Data Capture system (Medidata Institute, NY, USA). All statistical analyses were performed with SAS version 9.4 (SAS Institute, Inc., Cary, NC, USA).

Virus culture was previously planned as an exploratory endpoint in this study’s protocol. For the quantification of SARS-CoV-2, nasopharyngeal swab samples were taken at predetermined intervals. All the clinical samples were transferred to a single central laboratory and were analyzed there. SARS-CoV-2 culture was performed and viral titers were assessed as described previously (17).
