# Supplementary material for: A Double-Blind, Randomized, Placebo-Controlled, Phase II Clinical Study To Evaluate the Efficacy and Safety of Camostat Mesylate (DWJ1248) in Adult Patients with Mild to Moderate COVID-19
Source: Antimicrob Agents Chemother. 2022 Dec 14;67(1):e00452-22. doi: 10.1128/aac.00452-22 (PMC9872678; doi:10.1128/aac.00452-22)
Supplement: Supplemental file 1 — Supplemental material. Download aac.00452-22-s0001.pdf, PDF file, 0.5 MB [file aac.00452-22-s0001.pdf]

Supplementary Table 1. Demographic and Baseline (Randomized Set) [Subgroup: ≥60 years or Risk Factor - Yes]

	DWJ1248 (N=116)	Placebo (N=112)	Total (N=228)
**Smoker, n(%)**	116	112	228
Non-Smoker	67 (57.76)	68 (60.71)	135 (59.21)
Smoker	25 (21.55)	25 (22.32)	50 (21.93)
Ex-Smoker	24 (20.69)	18 (16.07)	42 (18.42)
Unknown	0 (0.00)	1 (0.89)	1 (0.44)
p-value [£]			0.6821 (f)
**Risk factor, n(%)**	116	112	228
Yes	96 (82.76)	90 (80.36)	186 (81.58)
No	20 (17.24)	22 (19.64)	42 (18.42)
p-value [£]			0.6400 (c)
**Cardiovascular disease1), n(%)**	96	90	186
Yes	13 (13.54)	11 (12.22)	24 (12.90)
No	83 (86.46)	79 (87.78)	162 (87.10)
**Chronic respiratory disease1), n(%)**	96	90	186
Yes	1 (1.04)	2 (2.22)	3 (1.61)
No	95 (98.96)	88 (97.78)	183 (98.39)
**High blood pressure1), n(%)**	96	90	186
Yes	50 (52.08)	54 (60.00)	104 (55.91)
No	46 (47.92)	36 (40.00)	82 (44.09)
**Diabetes1), n(%)**	96	90	186
Yes	28 (29.17)	19 (21.11)	47 (25.27)
No	68 (70.83)	71 (78.89)	139 (74.73)
**Obesity1), n(%)**	96	90	186
Yes	14 (14.58)	13 (14.44)	27 (14.52)
No	82 (85.42)	77 (85.56)	159 (85.48)
**Smoking1), n(%)**	96	90	186
Yes	25 (26.04)	25 (27.78)	50 (26.88)
No	71 (73.96)	65 (72.22)	136 (73.12)
**COVID-19 Severity at Baseline, n(%)**	116	112	228
Mild	89 (76.72)	71 (63.39)	160 (70.18)
Moderate	27 (23.28)	41 (36.61)	68 (29.82)
Severe	0 (0.00)	0 (0.00)	0 (0.00)
p-value [£]			0.0278 (c)
**COVID-19 Antibody, n(%)**	24	27	51
No	24 (100.00)	25 (92.59)	49 (96.08)
Yes	0 (0.00)	2 (7.41)	2 (3.92)
p-value [£]			0.4918 (f)
**Age group / Risk Factor, n(%)**	116	112	228
< 60years and Risk Factor - No	0 (0.00)	0 (0.00)	0 (0.00)
≥ 60years or Risk Factor - Yes	116 (100.00)	112 (100.00)	228 (100.00)

p-value [£]			NC
**Subjective Symptoms by Subject at Baseline, n(%)**	114	109	223
At least one Moderate or Severe	38 (33.33)	42 (38.53)	80 (35.87)
Other	76 (66.67)	67 (61.47)	143 (64.13)
p-value [£]			0.4185 (c)
**RT-PCR at Baseline, n(%)**	116	112	228
Inconclusive	1 (0.86)	0 (0.00)	1 (0.44)
Negative	3 (2.59)	2 (1.79)	5 (2.19)
Positive	112 (96.55)	110 (98.21)	222 (97.37)
p-value [£]			1.0000 (f)
**Ordinal scale at Baseline, n(%)**	116	112	228
0	2 (1.72)	2 (1.79)	4 (1.75)
1	0 (0.00)	0 (0.00)	0 (0.00)
2	46 (39.66)	45 (40.18)	91 (39.91)
3	68 (58.62)	65 (58.04)	133 (58.33)
p-value [£]			1.0000 (f)
**NEWS score at Baseline, n(%)**	116	112	228
0	50 (43.10)	47 (41.96)	97 (42.54)
1	39 (33.62)	39 (34.82)	78 (34.21)
2	19 (16.38)	15 (13.39)	34 (14.91)
3	6 (5.17)	8 (7.14)	14 (6.14)
4	1 (0.86)	3 (2.68)	4 (1.75)
5	1 (0.86)	0 (0.00)	1 (0.44)
p-value [£]			0.8067 (f)
**NEWS Severity at Baseline, n(%)**	116	112	228
Mild	115 (99.14)	110 (98.21)	225 (98.68)
Moderate	1 (0.86)	2 (1.79)	3 (1.32)
Severe	0 (0.00)	0 (0.00)	0 (0.00)
p-value [£]			0.6168 (f)

SD = standard deviation, Min = minimum, Max = maximum, NC = not calculated.

Note: Denominator of percentage is the number of subjects in each group.

[†] Testing for difference between treatment groups (two sample t-test (t) or Wilcoxon rank-sum test (w)).

[£] Testing for difference among treatment groups (chi-square test (c) or Fisher's exact test (f)).

Supplementary Table 2. Concomitant Medication (Randomized Set)

	DWJ1248 (N=172)	Placebo (N=170)	Total (N=342)
**Subjects with Concomitant Medication**	149(86.63) [1706]	157(92.35) [1701]	306(89.47) [3407]
95% Confidence Interval	[81.54, 91.71]	[88.36, 96.35]	[86.22, 92.73]
p-value [£]			0.0845 (c)
**Subjects with Analgesic**	113(65.70) [241]	115(67.65) [250]	228(66.67) [491]
95% Confidence Interval	[58.60, 72.79]	[60.61, 74.68]	[61.67, 71.66]
p-value [£]			0.7022 (c)
**Subjects with Anti**	55(31.98) [80]	47(27.65) [69]	102(29.82) [149]
95% Confidence Interval	[25.01, 38.95]	[20.92, 34.37]	[24.98, 34.67]
p-value [£]			0.3815 (c)
**Subjects with AntiHistamine**	46(26.74) [53]	45(26.47) [74]	91(26.61) [127]
95% Confidence Interval	[20.13, 33.36]	[19.84, 33.10]	[21.92, 31.29]
p-value [£]			0.9543 (c)
**Subjects with NSAIDs**	44(25.58) [80]	48(28.24) [74]	92(26.90) [154]
95% Confidence Interval	[19.06, 32.10]	[21.47, 35.00]	[22.20, 31.60]
p-value [£]			0.5800 (c)
**Subjects with Cough**	104(60.47) [340]	117(68.82) [373]	221(64.62) [713]
95% Confidence Interval	[53.16, 67.77]	[61.86, 75.79]	[59.55, 69.69]
p-value [£]			0.1060 (c)
**Subjects with Sore.T**	25(14.53) [33]	32(18.82) [42]	57(16.67) [75]
95% Confidence Interval	[9.27, 19.80]	[12.95, 24.70]	[12.72, 20.62]
p-value [£]			0.2873 (c)
**Subjects with Ms.Relax**	7(4.07) [14]	5(2.94) [5]	12(3.51) [19]
95% Confidence Interval	[1.12, 7.02]	[0.40, 5.48]	[1.56, 5.46]
p-value [£]			0.5706 (c)
**Subjects with Patch**	9(5.23) [12]	9(5.29) [12]	18(5.26) [24]
95% Confidence Interval	[1.90, 8.56]	[1.93, 8.66]	[2.90, 7.63]
p-value [£]			0.9797 (c)
**Subjects with Migraine**	4(2.33) [5]	0	4(1.17) [5]
Exact 95% Confidence Interval	[0.64, 5.85]	[0.00, 2.15]	[0.32, 2.97]
p-value [£]			0.1228 (f)

Note: Denominator of percentage is the number of subjects in each group.

Results are displayed as 'number of subjects(percentage of subjects) [number of event]'.

[£] Testing for difference among treatment groups (chi-square test (c) or Fisher's exact test (f)).

Supplementary Figure 1. Clinical improvement defined

< Classification of general severity>

Score	State	Classification of general severity
0	None	No symptoms
1	Mild	If it is easily tolerated by causing minimal discomfort without interfering with normal daily life
2	Moderate	If it causes discomfort that interferes with normal daily life to some extent
3	Severe	When it makes normal daily activities impossible

Example:

COVID-19 Symptoms		Baseline	Treatment period (day)													
		**Day 1**	**2**	**3**	**4**	**5**	**6**	**7**	**8**	**9**	**10**	**11**	**12**	**13**	**14**	**15**
a	Feeling hot	**2**	2	2	1	2	3	**1**	2	2	**1**	2	1	3	**1**	1
b	Cough	**2**	2	2	2	2	1	**0**	2	2	**0**	2	0	2	**0**	0
c	Sorthness of breath	**1**	0	0	0	0	0	**0**	0	0	**0**	1	0	1	**0**	0
d	Chills	0	0	0	0	0	0	0	0	0	0	0	0	0	0	0
e	Muscle ache	**1**	0	1	0	0	0	**0**	1	1	**0**	1	0	1	**0**	0
f	Headache	0	0	0	0	0	0	0	0	0	0	0	0	0	0	0
g	Sore throat	0	0	0	0	2	0	1	2	0	0	0	1	0	0	1

Subjects were identified as having COVID-19 disease and enrolled within 7 days of symptom onset according to our protocol inclusion criteria.

The subject had symptoms ‘a’, ‘b’, ‘c’ and ‘e’ on Day 1 (=baseline), thus was administrated an Investigational Product.

- On Day 4, symptoms ‘a’, ‘c’ and ‘e’ were improved, but symptom ‘b’ was maintained at 2 points, so there was no “clinical improvement”.
- On Days 7 symptoms ‘a’, ‘b’, ‘c’ and ‘e’ were improved, but symptom ‘g’ was worsened to 1 point, so there was no “clinical improvement”.
- On Day 10 and 14, symptoms ‘a’, ‘b’, ‘c’ and ‘e’ were improved, and all other symptoms maintained at 0 points, so there was “clinical improvement”.

Time to clinical improvement of subjective symptoms, defined as the first day of symptom improvement.
